# Multichannel full-space coding metasurface with linearly-circularly-polarized wavefront manipulation

**DOI:** 10.1515/nanoph-2024-0331

**Published:** 2024-08-29

**Authors:** Huiling Luo, Huanhuan Gao, Yanzhao Wang, Chaohui Wang, Fan Zhang, Yanzhang Shao, Tong Liu, Zhengjie Wang, He-Xiu Xu

**Affiliations:** Air and Missile Defense College, Air Force Engineering University, Xi’an 710051, China

**Keywords:** multichannel coding metasurface, full space, wavefront control, shared aperture, linearly-circularly-polarized

## Abstract

Achieving independent multitasked wavefront control by using an ultrathin plate is a challenge to increase information capacity in integration optics and radar applications. Transmission-reflection-integrated metasurface provides an efficient recipe primarily for multifunctional meta-device, however it is challenging to synergize both linear polarization (LP) and circular polarization (CP) using a single meta-plate. Here, a multichannel full-space coding metasurface composed of interleaved shared-aperture meta-atom is proposed to achieve large information capacity by capsulating judiciously engineered high efficiency triple sub-elements (modes) in four-layer scheme. By rotating dual-gap split ring resonator and varying size of “L” type structure insulating by a metallic ring with electrostatic-analogue shielding effect, both Pancharatnam–Berry (PB) and dynamic phases are independently realized under CP and LP waves, respectively. Such an extraordinary insulating strategy completely suppresses crosstalk among three modes and unprecedentedly increases the capability in yielding kaleidoscopic wavefront control. To verify the significance, a proof-of-concept metadevice is devised and experimentally demonstrated with tri-channel wavefront manipulations, exhibiting reflective dual-vortex beam and Bessel beam for forward and backward CP wave, respectively at high frequency, while transmissive polarization beam splitting for 45°-LP wave at low frequency. Our finding in polarization-direction multiplexing is expected to generate great interest in electromagnetic integration with emerging degree of freedoms.

## 1 Introduction

As a planar version of artificial metamaterial, metasurface exhibits unprecedented ability to precisely control phase, amplitude, and polarization of electromagnetic (EM) waves by introducing field discontinuities across an interface [1], [2], [3], [4], [5], [6] in response to actual industrial demand. To date, metasurfaces have been widely applied to polarization conversion [7], [8], radar cross section (RCS) reduction [9], anomalous refraction or reflection [10], [11], [12], orbital angular momentum (OAM) generation [13], [14], [15], holography generation [16], [17], multibeam transmitarray [18], retroreflector [19] and beam diffraction management [20]. To bridge the gap between the physical realization and information science [21], the concept of coding metasurfaces was initially proposed by Cui’s group in 2014. Since then, metasurfaces have been further developed in physical world, engineered with pre-designed coding sequences and physical array topology [22], [23]. However, most of them are confined to mono-function and thus cannot meet the requirements of modern integration systems, which may seriously hinder applications of resulting metadevices.

With the rapid development of future communication, multifunction metasurfaces have been developed to meet the increasing demands of high-capacity systems and devices by integrating multiple information channels over a single meta-plate [24]. To date, they are generally classified into two types (active and passive metasurfaces) based on whether or not active components are utilized. Although active metasurfaces have been proposed to dynamically achieve various functions through reconfigurable [25], [26], [27], [28], [29], [30] and programmable [31], [32], [33] techniques, there still exists several challenges including complex fabrication, high cost and high insertion loss, and thus limit their applications. Thus, multifunctional passive metasurfaces is a promising candidate to tackle above challenges. To further expand the channel capacity, new perspectives of multiplexing techniques have been explored by combining two or more degrees of freedom (DoFs) [34], [35], [36], [37], [38], [39], [40], [41], [42], such as polarization [43], frequency [44], [45], angular [46], space [47], [48] and direction [49] multiplexing. Among them, transmission-reflection-integrated multifunctional metasurfaces are one of the most representative owing to their extraordinary capability of manipulating EM waves in full space. To implement these in microwave band, some special schemes have to be introduced, such as frequency-selective metallic plane for frequency multiplexing [38], [39], [40], [41], [50], [51], [52], slotted ground plane for direction multiplexing [38] and a metallic via-hole connecting receiver and transmitter patches for polarization multiplexing [53], [54]. However, to the best of our knowledge, most of previous attempts are limited to either linear polarization (LP) or circular polarization (CP) operations, while full-space wavefront control with combined LP and CP channels in a shared-aperture scheme is rarely reported.

In light of aforementioned issues, here, we report a paradigm of synergizing polarization-direction multiplexing with PB and dynamic phases modulations in a tri-channel metasurface with full-space manipulations of EM waves. By separately rotating the bottom and top dual-gap split ring resonators (SRRs) in two reflection channels, two independent phase shifts are attained for CP wave along −*z* and +*z* directions, respectively. Meanwhile, varying size of “L” type structures in a transmission channel achieve polarization beam splitting under LP wave excitation due to independent phase shifts. Most importantly, the channel interference or polarization crosstalk therein is smartly suppressed by the shielding effect of a metallic ring. Our strategy improves the utilization of space resources and opens up an alternative avenue for meta-devices with unprecedented data capacity, which shows particularly relevant for potential applications in EM control.

## 2 Results and discussion

### 2.1 Concept and meta-atom design

**Figure 1:**
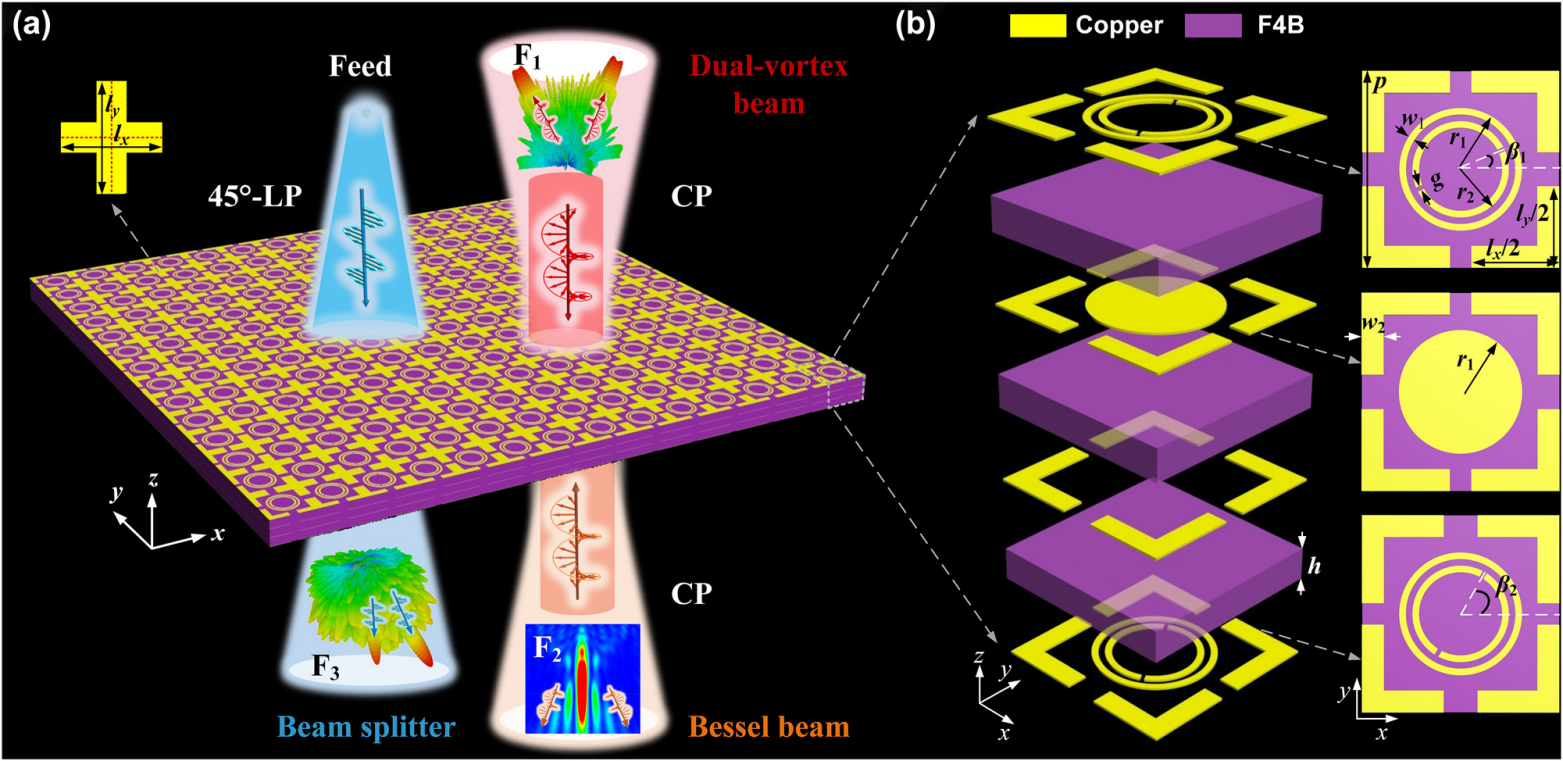
Schematic and working principle of proposed multichannel full-space coding metasurface with linearly-circularly-polarized wavefront manipulation. (a) Multichannel coding metasurface, *F*_1_, *F*_2_ and *F*_3_, denoting three independent functionalities of dual-vortex beam launcher, Bessel beam launcher and polarization dependent beam splitter, respectively. (b) Design and characterization of the proposed coding meta-atom. Here, each layer of the metallic pattern exhibits four “L” type patches in every corner, which forms a diagonal crossbar patch with those of three adjacent meta-atoms in ultimate metasurface.

Figure 1(a) illustrates the conceptual diagram of the proposed multichannel full-space coding metasurface. Depending on the multiplexing of polarizations and directions of incident waves, our engineered metasurface works under both reflective and transmissive modes, independently performing different functions (*F*_1_, *F*_2_, *F*_3_) in LP and CP channels. In two reflective cases, the forward (+*z*-axis) and backward (−*z*-axis) incoming CP wave was transformed into dual-vortex beam (*F*_1_) at 17.1 GHz and anomalous deflection (*F*_2_) at 16.9 GHz, respectively, whereas for transmissive case the backward 45°-LP wave excited by feeding horn can be split into *x*- and *y*- LP beams (*F*_3_) at 11.2 GHz.

To engineer this concept, the trifunctional metadevice is comprised of size-varied and orientation-rotated composite meta-atoms. The devised meta-atom is composed of four metallic layers and three F4B substrates (*ε*_*r*_ = 2.65, *δ* = 0.001), and an interleaved shared-aperture structure is designed to implement linearly-circularly-polarized wavefront manipulation, see our designed magnified basic building block given in Figure 1(b). A dual-gap split ring resonator (SRR) is surrounded by a metallic ring in the center of both top and bottom layers, and the second-layer middle circle patch plays a role of the ground plane that can reflect EM wave. Each layer of the metallic pattern exhibits four “L” type patches in every corner, which forms a diagonal crossbar patch with those of three adjacent meta-atoms. Here, metallic rings suppress mode crosstalk, and the interlayer coupling between four-layer “L” type patches forms a Fabry–Perot resonance to enhance phase accumulation. To simplify design process, the basic meta-atom is roughly determined with several detailed geometric parameters as follows: *p* = 9 mm, *h* = 1.5 mm, *r*_1_ = 2.8 mm, *r*_2_ = 2.2 mm, *w*_1_ = 0.3 mm, *w*_2_ = 1 mm, and *g* = 0.2 mm.

**Figure 2:**
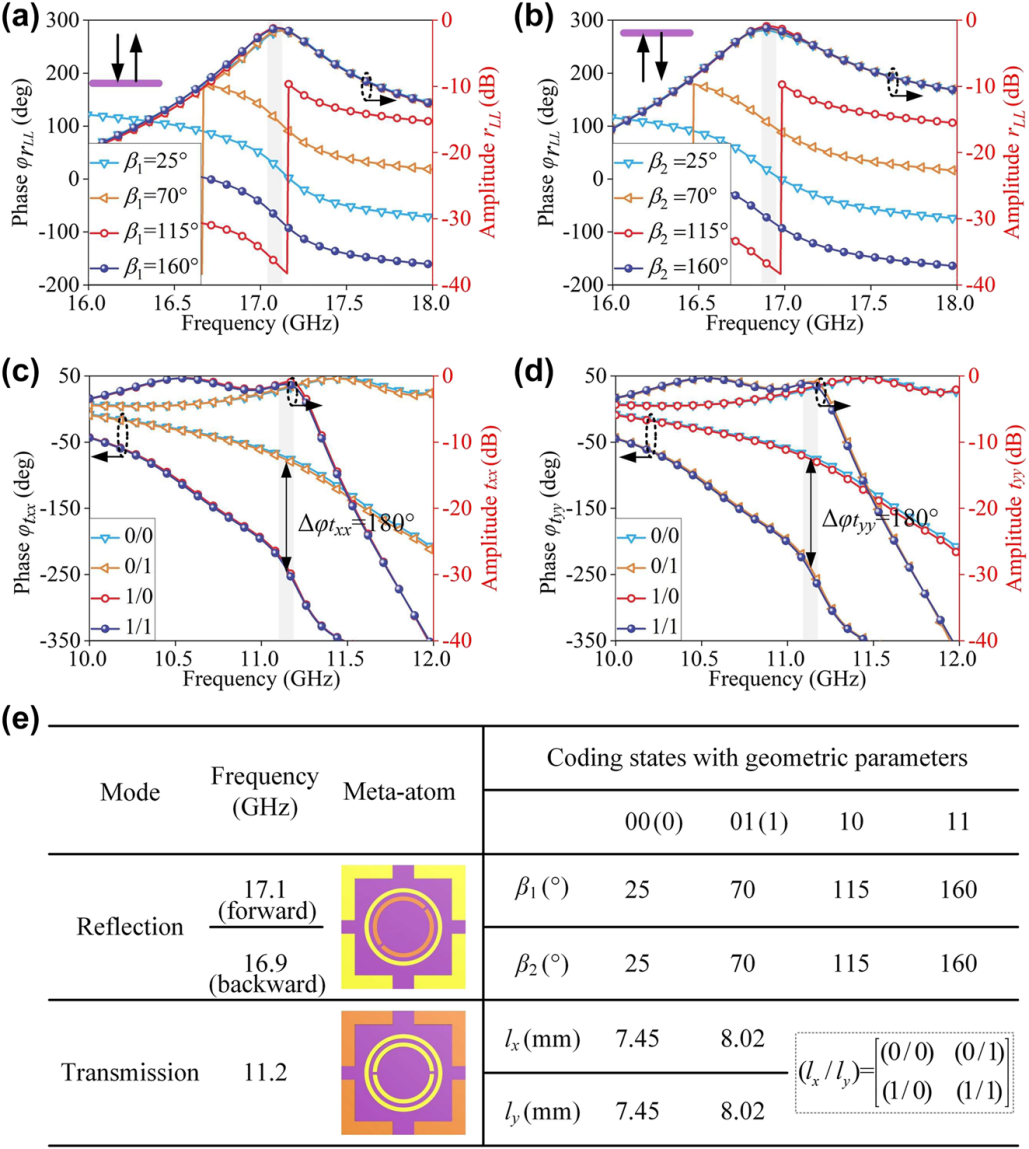
Reflection and transmission performance of the coding meta-atoms under CP and LP waves. (a, b) Reflection phase and amplitude with varying parameters of rotation angle *β*_1_ and *β*_2_ under incidence of LCP wave along −*z* direction at 17.1 GHz and +*z* direction at 16.9 GHz, respectively. (c, d) Transmission phase and amplitude with different size parameters *l*_*x*_ and *l*_*y*_ under incidence of *x*- and *y*- LP waves along −*z* direction at 11.2 GHz, respectively. (e) Specific dimensions and corresponding codes of two types of coding sub-meta-atoms.

The most important property of our polarization-direction multiplexed meta-atom is that EM response of reflection coding states is closely associated with rotation angles of *β*_1_, *β*_2_, while that of transmission coding states depends on geometric parameters *l*_*x*_ and *l*_*y*_. To demonstrate the feasibility of our strategy, full-wave finite-difference time-domain (FDTD) calculations are performed through CST Microwave Studio commercial packet. Firstly, reflection PB phase control is carried out for 2 bit coding states under LCP wave along −*z* and +*z* directions (identical electromagnetic properties are expected in RCP wave case according to the principle of conjugacy). By rotating dual-gap SRRs of the first or fourth layer, four rotational angles *β* are optimized as 25°, 70°, 115° and 160° to achieve four π/4 phase gradients. As illustrated in Figure 2(a) and (b), the relationship between extra phase shift Δ*φ* and rotation angle Δ*β* can be expressed as Δ*φ* = 2Δ*β* according to PB phase principle to ensure perfect linear phase shift. Therefore, the reflection π∕4 phase gradient of the four coding meta-atoms nearly covers 2π, corresponding to “00,” “01,” “10,” and “11.”, and the reflection amplitudes are all above −1.5 dB at 17.1 and 16.9 GHz with fixed geometric parameters. In addition, there is a slight operation frequency difference between forward and backward reflection channel due to different height of top (*h*) and bottom (2*h*) dual-gap SRRs to the second-layer circle patch.

Next, we consider the transmission case for dynamic phase control. Four sets of parameters are selected by adjusting and optimizing four-layer “L” type patches (*l*_*x*_ and *l*_*y*_) to achieve independent 1 bit phase manipulation for *x*- and *y*- LP incidence waves. The length *l*_*x*_ is optimized as 7.45 and 8.02 mm, which is the same as *l*_*y*_. The co-polarized transmissive amplitude and phase response of meta-atom under *x*- and *y*- LP waves from 10 GHz to 12 GHz are plotted in Figure 2(c) and (d). The lengths *l*_*x*_/*l*_*y*_ for the four coding states “0/0”, “0/1”, “1/0”, and “1/1” are optimized as 7.45 mm/7.45 mm, 7.45 mm/8.02 mm, 8.02 mm/7.45 mm, and 8.02 mm/8.02 mm, respectively. Two-digit number before and after the slash represents the coding size-sequence of the “L” type structure under *x*- and *y*- LP wave incidence, respectively. It can be seen that co-polarization transmissive amplitude is higher than −2 dB, and the phase difference between “0”-unit and “1”-unit is about 180° at 11.2 GHz. Noting that the amplitude and phase response under *x*- LP wave is determined only by *l*_*x*_, where a change in *l*_*y*_ barely affects it, and vice versa. The results above indicate very low EM coupling under two orthogonal *x*- and *y*- LP wave excitation at 11.2 GHz. Figure 2(e) summarizes detailed values of *β*_1_ and *β*_2_, and *l*_*x*_ and *l*_*y*_ corresponding to 2 bit/1 bit coding states. Namely, dual-layer dual-gap SRR are rotated and size of four-layer “L” type patches are varied, respectively in a single meta-atom, as shown in the dark yellow of inset of Figure 2(e). To distinguish two sets of coding states, the devised composite meta-atom is decomposed into two types of coding sub-meta-atoms individually for reflection and transmission.

**Figure 3:**
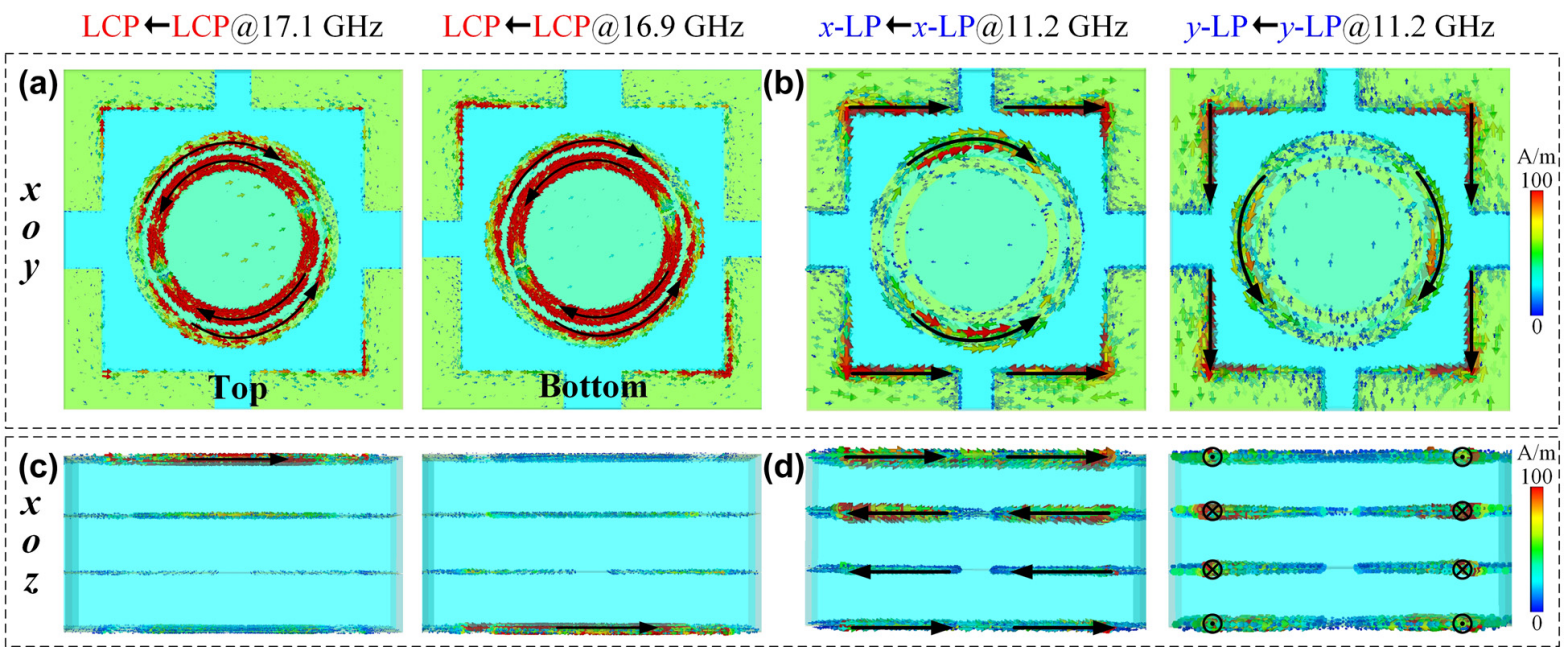
Simulated current distributions of the entire meta-atom. (a) Current distributions of top and bottom layers for backward and forward LCP wave excitation at 17.1 GHz and 16.9 GHz. (b) Top view of current distribution under *x*- and *y*-polarized waves incidence at 11.2 GHz. (c, d) Side view of corresponding current distribution.

The physical mechanism for independent control three sets of phase can be inspected from the current distributions at three modes shown in Figure 3(a) and (b). The loop currents are formed on the top dual-gap SRR and ring patch at 17.1 GHz when the designed metasurface is assumed to be illuminated by LCP plane wave propagating along −*z* direction, while they are strongly localized on the bottom dual-gap SRR and ring patch for LCP wave along +*z* direction at 16.9 GHz. In sharp contrast, when the meta-atom is illuminated by *x*- and *y*- LP waves at 11.2 GHz, the current mainly concentrates on “L” type and ring patches. Moreover, the current is formed on one face of the top and bottom metallic layers in two reflection channels at 17.1 and 16.9 GHz, respectively, whereas it is formed on four faces of the structure at 11.2 GHz in the transmission channel as portrayed in Figure 3(c) and (d). Specifically, Figure 3(d) show that the antiparallel loop currents are formed on two faces of the first and second (third and fourth) metallic layers under LP wave incidence at 11.2 GHz. This is strong evidence of induced magnetic effect contributing to the high efficiency transmission. In all cases, the current at residual parts is extremely weak, indicating that they are barely responding. Most importantly, the metallic rings exhibit shielding effect against external electric fields of “L” type patches for CP wave at high frequency and those of SRRs for 45°-LP wave at low frequency. Namely, the metallic ring acts as an electrostatic shield against external electric fields, reducing the crosstalk between LP and CP modes, similar to electrostatic shielding effect in physics. Therefore, the metallic ring ensures the phase response under LP and CP waves independently modulated by rotating dual-gap SRRs and varying size of “L” type patches, respectively. Thus, the designed composite mate-atom essentially reduces internal mutual coupling, providing good isolation and allowing independent tri-channel wavefront control.

**Figure 4:**
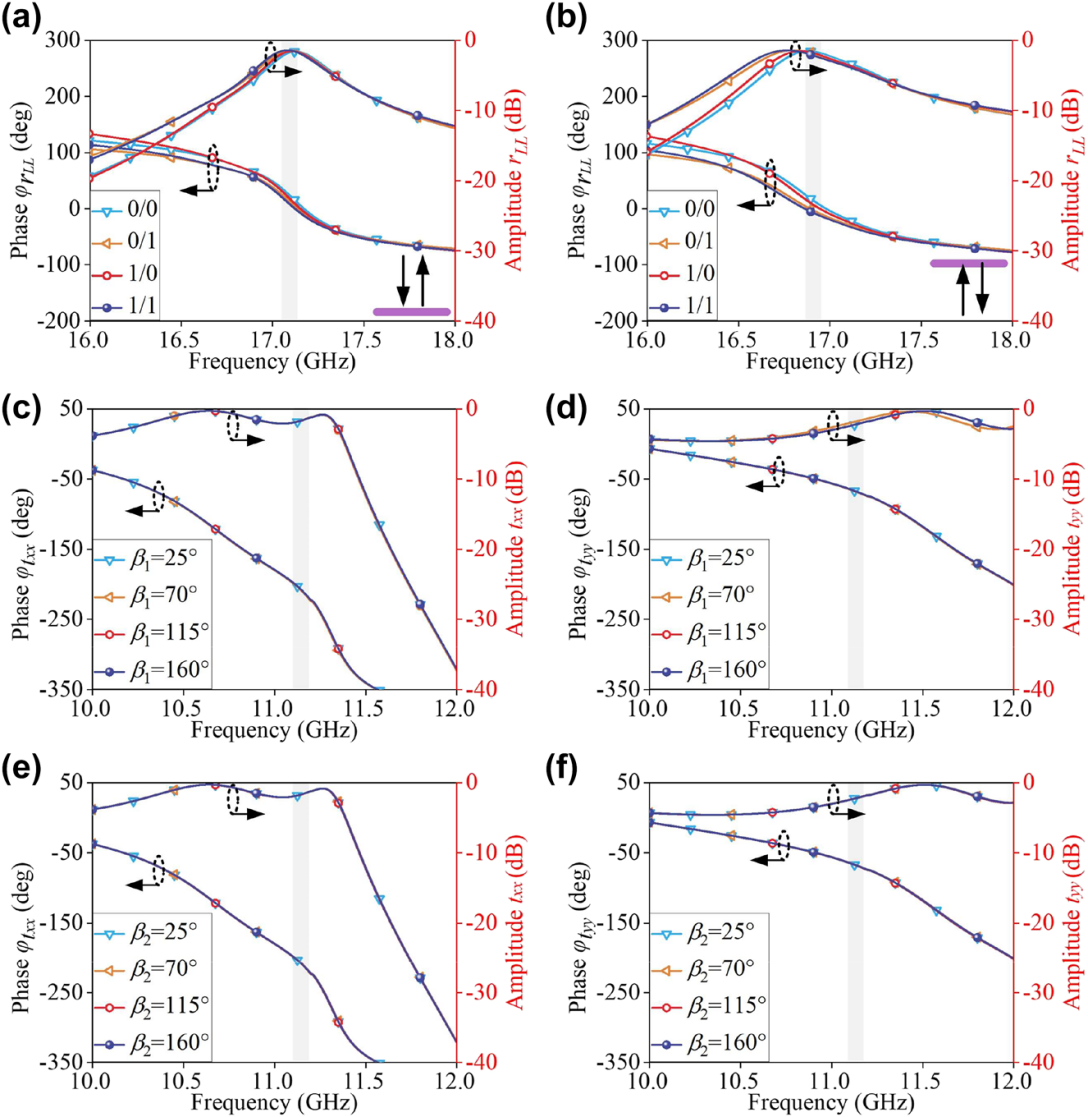
Coding states crosstalk. (a, b) 2 bit coding states with reflection amplitude and phase response under different values of *l*_*x*_ and *l*_*y*_, when *β*_1_ and *β*_2_ are fixed as 25° and 70° at 17.1 and 16.9 GHz, respectively. 1 bit coding states with transmission amplitude and phase response under different values of rotation angle (c, d) *β*_1_ and (e, f) *β*_2_, when *l*_*x*_ and *l*_*y*_ are specified as 7.45 and 8.02 mm.

To directly validate minimal crosstalk among modes at aforementioned three channels, Figure 4 shows the FDTD-calculated amplitude and phase responses of 2 bit coding states in forward and backward reflection cases while those of 1 bit coding states in transmission case. Figure 4(a) and (b) show that changing *l*_*x*_ and *l*_*y*_ have negligible influence on the reflection response under CP wave along *x*- and *y*-directions. As depicted in Figure 4(c)–(f), the transmission response is solely determined by changing *l*_*x*_ and *l*_*y*_ of the “L” type patches under the *x*- and *y*- LP waves at 11.2 GHz, while it is hardly affected by rotating *β*_1_ and *β*_2_ of SRRs. Most importantly, the consistency of the EM response indicates a low cross-polarization level. Moreover, we further explore the role of designed metallic ring by comparing coupling level of meta-atom without metallic ring at its corresponding frequencies, see Figure S1 (Supporting Information). Thus, channel interference is smartly suppressed by the shielding effect of a metallic ring under LP and CP wave, which affords a solid platform for full-space and tri-channel wavefront control. Based on above analyses, it can be concluded that PB phase control of reflection channel is achieved by rotating dual-gap SRRs under CP wave, and dynamic phase control of transmission channel is achieved by varying size of “L” type patches under LP wave.

### 2.2 Metadevice design and numerical results

To further validate potential applications of proposed coding meta-atom, we conceive a trifunctional full-space metadevice through 2 bit reflection and 1 bit transmission coding sub-meta-atoms. The metadevice is composed of 32 × 32 composite meta-atoms, which occupies an area of 288 × 288 mm^2^. Here, four “L” type patches of each meta-atom in resulting array are different depending on on-demand functions, and are ultimately formed as crossbar patches in diagonal to dual-gap SRR, see Figure 1(a). Remarkably, the shared-aperture metastructure exhibits two optical axis located at center of the two types of patches, where independent wavefront control in LP and CP channels are realized based on adjusting sizes of crossbar patches and rotating dual-gap SRR, respectively.

**Figure 5:**
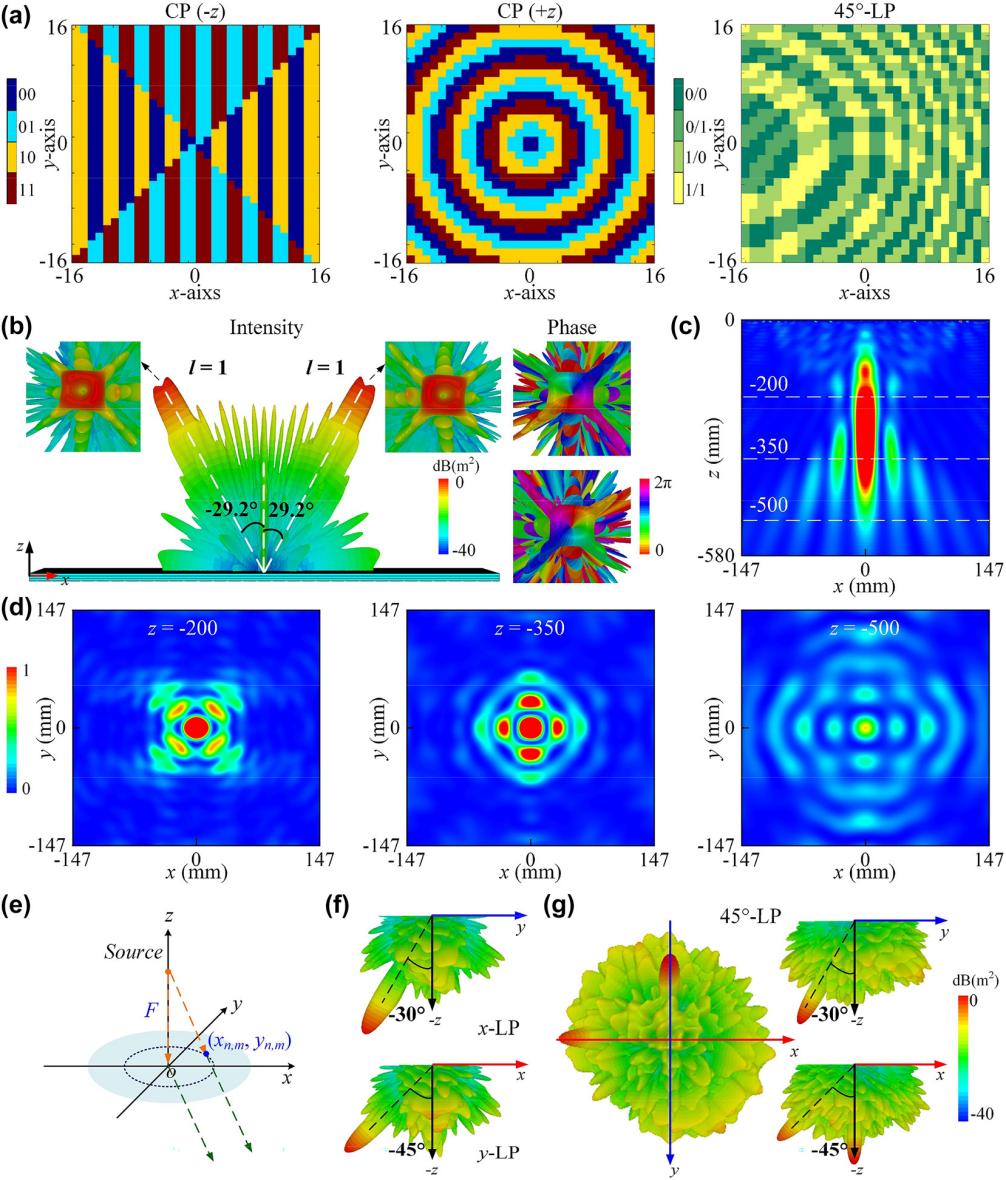
Simulated reflection and transmission performance of proposed full-space coding metasurface. (a) Coding patterns of tri-channel metadevice with dual-vortex beam, Bessel beam, polarization beam splitting. (b) 3D far-field intensity and phase patterns under illumination of backward CP wave at 17.1 GHz in reflection region. Near-field intensity on (c) *x*–*z* plane (d) and *x*–*y* planes of *z* = 200, 350, 500 mm under illumination of forward CP wave at 16.9 GHz in reflection region. (e) The corresponding metasurface schematic. Simulated 3-D radiation pattern under illumination of (f) *x*-LP, *y*-LP and (g) 45°-LP waves at 11.2 GHz in transmission region.

It is worth noting that diverse beam shaping stems from pre-designed coding sequences. For example, we design a tri-channel metadevice combined LP and CP modes, exhibiting dual-vortex beam, Bessel beam and polarization dependent beam splitting (*F*_1_, *F*_2_, *F*_3_). Combining the phase theoretical calculations with the coding states of geometric parameters from Figure 2(e), three coding patterns of tri-channel metasurface are shown in Figure 5(a). For CP wave along −*z*-axis, a dual-vortex beam is engineered along the *x* direction (detailed in Figure S2, Supporting Information), where its synthesized sequence is obtained by convolution theorem of vortex beam carrying topological charge *l* = +1 and gradient sequences. Here, phase distribution of vortex beam is mathematically written as $\varphi_{1}=l\cdot \text{arc}\;\tan\left(y/x\right)$ , gradient coding sequence is encoded as “00001010 … ” along *x*-direction that can generate two scattered beams with deflection angle *θ* = ±29.2° (the phase distribution of scattered beam be described as *φ*_2_ = *k*_0_ × *x* × sin*θ*). For CP wave along +*z*-axis, the phase distribution of zero-order Bessel beams is described as: $\varphi_{3}=\frac{2\pi }{\lambda }\sqrt{x^{2}+y^{2}}\sin\alpha$ , where *α* = 15° is base angle of equivalent axicon, *λ* is wavelength. As to the transmission case at low frequency, *x*-LP wave along *y*-axis and *y*-LP wave along *x*-axis are split when 45°-LP feed source emits EM waves to the metasurface (detailed in Figures S3 and S4, Supporting Information). Thus, the synthesized phase distribution of polarization beam splitter is calculated by convolution theorem of phase $\phi_{y}\left(\theta_{y}\right)$ and $\phi_{x}\left(\theta_{x}\right)$ of two transmission beams. Here, $\phi_{y}\left(\theta_{y}\right)=k_{0}S_{n,m}-k_{0}\cdot y_{n,m}\sin\theta_{y}$ and $\phi_{x}\left(\theta_{x}\right)=k_{0}S_{n,m}-k_{0}\cdot x_{n,m}\sin\theta_{x}$ , where *k*_0_ = 2π*f*_0_ is the wave number in the free space at target frequency *f*_0_, $S_{n,m}=\sqrt{x_{n,m}^{2}+y_{n,m}^{2}+F^{2}}$ is the distance transmission from the (*n*, *m*)th meta-atom to the feed source.

To further analyze near-field and far-field characteristics, integrated metasurface mode with open (add space) boundary conditions in all directions is used in CST Microwave Studio. We first discuss two functions (*F*_1_ and *F*_2_) under forward and backward CP waves excitation. Vortex beams carrying OAM have attracted great interest in the last decades due to their ability to carry more information in a set of inherently insulated channels among mutually orthogonal modes in EM fields. Therefore, the generation of dual-vortex beams is also important for wireless communication systems due to the necessity of large capacity. As is shown in Figure 5(b), we validate the simulated 3D far-field result of dual-vortex beams (*F*_1_) under normal illumination of CP incident wave along −*z* axis at 17.1 GHz. It can be seen that two generated beams exhibit ring-shaped intensity profile with the hollow center, and the intensity null of each beam is nearly located at the pre-designed beam deflection angle, which is consistent with the characteristics of the dual-vortex beam. Specifically, a 2*π* spiral phase is accumulated in each beam during one clockwise revolution, indicating dual OAM beams with modes of *l* = 1. Bessel beam generation (*F*_2_), as a classical non-diffracting beam, possesses inherent advantages of long energy transmission distance and wide focusing range, which are widely used in optical systems. As shown in Figure 5(c), the calculated near-field intensity in *x*–*z* plane shows a non-diffraction beam with excellent performance in CP channel along +*z* axis at 16.9 GHz. Furthermore, corresponding reflection electric field intensity in *x*–*y* plane at *z* = 200, 350 and 500 mm above the metaplexer is illustrated in Figure 5(d), where a bright maximum appears in the central, exhibiting a good zero-order Bessel beam. Meanwhile, we plot energy distribution of Bessel beam along the *x*-axis at *y* = 0 to intuitively show the energy variation in above three *x*–*y* planes, as shown in Figure S5 (Supporting Information).

In the following, we demonstrate transmitted function *F*_3_. Polarization dependent beam splitter, as a typical component controlling the differently polarized waves independently, has been found of great importance in both optical and microwave regimes. To suppress side-lobes in our design, the metasurface is excited by an LP horn at a distance of *F* = 150 mm, which guarantees high radiation beam generation, see Figure 5(e). To provide an intuitive view of the novel beam splitting, Figure 5(f) and (g) show the 3D far-field patterns under illumination of differently polarized waves at 11.2 GHz. From the radiation pattern in Figure 5(f), the deflected beams are clearly observed along *y*-direction with *θ*_*y*_ = −30° and *x*-direction with *θ*_*x*_ = −45° when the metadevice is excited by the *x*- and *y*- LP waves, respectively, demonstrating an excellent agreement with theoretical prediction. To verify the polarization-independent property, simulated results excited by 45°-LP wave are shown in Figure 5(g). As expected, two separated beams at *x*-direction with *θ*_*x*_ = −45° and *y*-direction with *θ*_*y*_ = −30° are observed, which are essentially same as the radiated beams of Figure 5(f), indicating excellent polarization splitting performance. In summary, the well-designed polarization beam splitter exhibits different polarization responses and is capable to split the orthogonally polarized wave completely.

### 2.3 Experimental results and discussion

For experimental demonstrations under differently polarized waves, a proof-of-concept meta-device prototype is fabricated using printed circuit board (PCB) technology, as shown in Figure S6 (Supporting Information). The outer copper on the surface is tinned to protect the sample from being oxidized. Furthermore, four-layer metallic patterns are printed individually on three F4B dielectric boards, and then assembled through adhesives and reinforced through a hot press. Next, we characterize the performance of the tri-channel transmission-reflection-integrated meta-device in near-field and far-field experiments.

**Figure 6:**
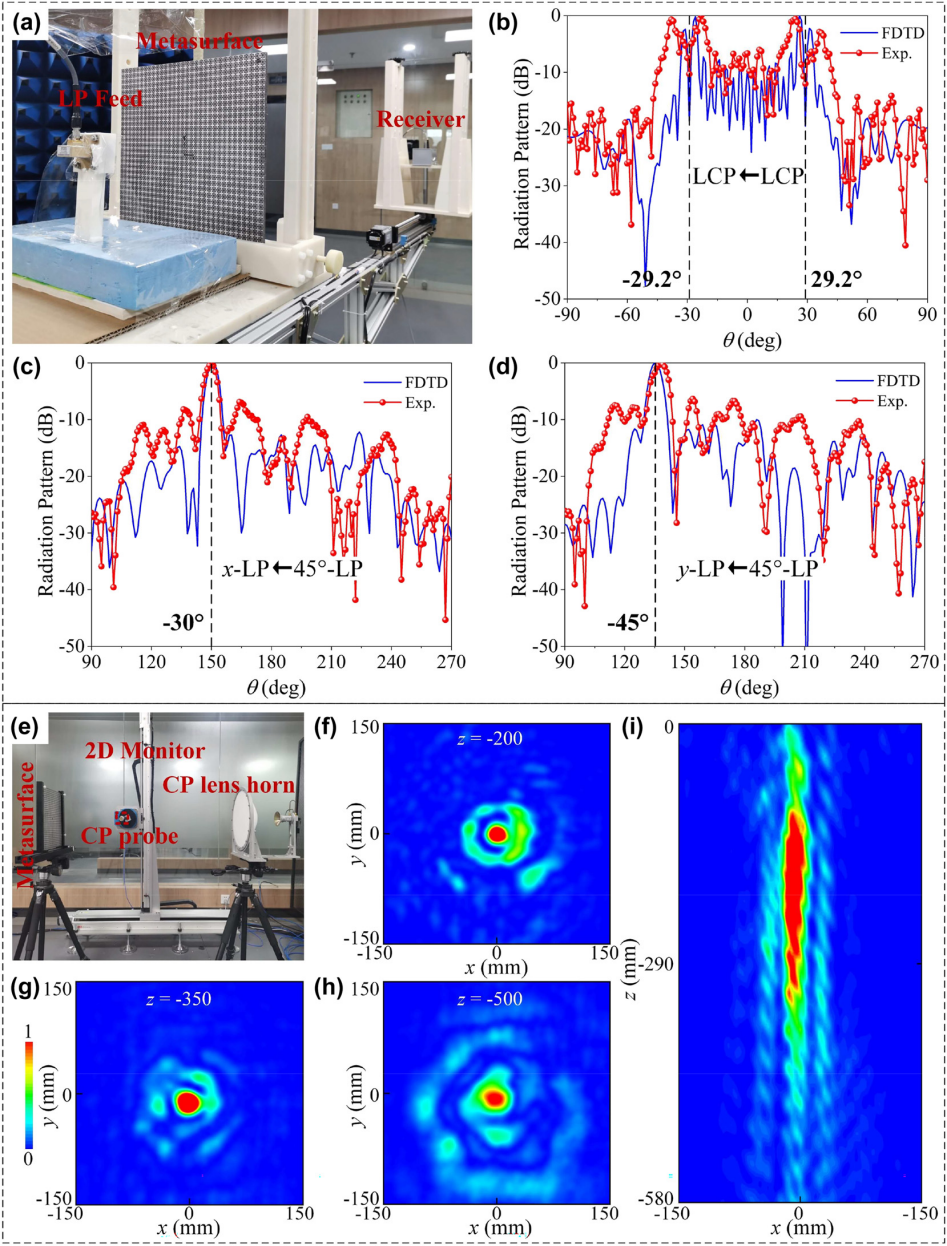
Far-field and near-field experimental demonstration of full-space coding metadevice. (a) Far-field experimental setup. (b) FDTD simulated and measured reflection radiation patterns under illumination of LCP wave along +*z* direction at 17.1 GHz. (c, d) FDTD simulated and measured transmission radiation patterns under illumination of 45°-LP wave at 11.2 GHz. (e) Near-field experimental setup. Measured near-field intensity on (f–h) *x*–*y* planes of *z* = 200, 350, 500 mm and on (i) *x*–*z* plane under illumination of CP wave along +*z* direction at 16.9 GHz. The scanning areas of *x*–*y* and *x*–*z* planes are ≈0.3 m × 0.3 m and 0.3 m × 0.58 m in steps of 5 mm, respectively.

To gain a straightforward understanding of the dual-vortex beam and polarization beam splitting, far-field measurements are conducted, and the experimental setup is shown in Figure 6(a). The transmitted horn (broadband dual-CP horn working at 8–18 GHz/small-sized LP feeding horn working at 8–12 GHz) is mounted on a fixed plate. Meanwhile, the received horn (broadband dual-CP horn/LP horn) is attached to an automatically rotated arm, which is controlled by an electronic motor to move around the sample in a circular motion. All EM signals are recorded through an AV3672B vector network analyzer. From Figure 6(b)–(d), it is indicated that the measured scattering beams indicated by the red dot line cover exactly FDTD calculated ones denoted by the blue solid line, verifying the rationality of our design. To investigate the dual-vortex beam, we first characterize the simulated and measured results when impinged by a normally incident LCP wave at 17.1 GHz, see Figure 6(b). Two measured helical beams in the reflection region are directed approximately at −29.2° and 29.2°, and the energy null of each beam is nearly located at the pre-designed beam deflection angle. Most importantly, the measured specular scattering between two anomalous main lobes is relatively low and is observed near −10 dB, indicating an excellent dual-vortex beam. In the case of transmission, the function of polarization beam splitter is excited by 45°-LP wave with the feeding horn antenna rotating 45° in alignment with the electric field. The radiation patterns of splitting *x*- and *y*- LP waves at 11.2 GHz are further depicted in Figure 6(c) and (d). It is seen clearly that the deflected angles are −30° and −45° in *y*–*z* and *x*–*z* planes, respectively, which are consistent with theoretical ones. In addition, the radiation patterns under illumination of *x*- and *y*- LP waves are shown in Figure S7 (Supporting Information). Comparing 3D far-field patterns under illumination of differently polarized waves, it is evident that multichannel full-space coding metasurface exhibits good polarization splitting performance. The slight disagreement in the shapes of the main lobe and sidelobes is mainly due to the imperfect matching of the measurement environment and the fabrication errors. Overall, the measured results show good agreement with the numerical ones, which proves the feasibility of our design. Furthermore, the efficiency of two beam deflection is simulated as 89.3 % and 88.5 %, and measured as 87.2 % and 85.1 %, respectively.

The 2D near-field scanning measurements are performed to explore the performance of Bessel beam generation, as illustrated in Figure 6(e). The fabricated sample is excited by an LCP horn from backward incidence, and the LCP field is probed by small-aperture LCP spiral antennas (*D* = 20 mm). Here, to afford a rapid plane-wave excitation without much divergence of antenna fields, a broadband dual-CP dielectric lens horn antenna with an aperture of 280 mm and an axial ratio of less than 3.5 dB within 6–18 GHz is utilized as a transmitter. Next, by varying the position of the probe via the motion controller, *x*–*y* and *x*–*z* planes can be covered and experimental field intensities can be measured. Such a good performance is also supported by measurement results, see Figure 6(f)–(i). As shown in Figure 6(f)–(h), a bright spot with maximum energy is clearly observed at the center of *x*–*y* planes of *z* = 200, 350 and 500 mm, respectively, verifying a long energy propagation Bessel beam. As can be seen from Figure 6(i), the energy in the *x*–*z* plane is mainly confined around the optical axis (*z*-axis), indicating a clear nondiffractive propagation of Bessel beam. Slight deviations between simulation and experiment are partially attributable to unavoidable errors in measurements due to unexpected arrangements of the sample and exciting horn, and partially to tolerances inherent in PCB fabrication.

## 3 Conclusions

To sum up, we have proposed and experimentally demonstrated a multifunctional full-space coding metasurface capable of simultaneously controlling of the reflected and transmitted wavefronts in polarization-direction multiplexing. By elaborately arranging specific coding sequences at corresponding modes, a super metadevice is devised to integrate three distinct functions on a shared-aperture configuration. Furthermore, the proposed method will get rid of the in-band interference and suppress polarization cross-talk by introducing the metallic ring with electrostatic-analogue shielding to improve the information capacity distinctly. Experimental results have verified all predesigned functions with excellent performance, showcasing dual-vortex beam launcher, Bessel beam launcher and polarization dependent beam splitter, respectively. Our proposed full-space multitask metaplexer opens new possibilities to promote the information capacity of coding metasurface and paves the way to multitasking systems, promising great potential applications in the microwave and photonic applications in the future.

## Supporting Information

Comparison for coupling level of the meta-atom with and without metallic rings in the top and bottom layers; The design process of coding pattern for dual-vortex beam; Transmission phase calculation and coding pattern of polarization dependent beam splitting; FDTD calculated field intensity of Bessel beam at *x*–*z* planes; Photograph of fabricated sample; Transmission radiation patterns under illumination of *x*- and *y*-LP waves at 11.2 GHz.

## Supplementary Material

Supplementary Material Details
