# Supplementary material for: Multichannel full-space coding metasurface with linearly-circularly-polarized wavefront manipulation
Source: Nanophotonics. 2024 Aug 29;13(22):4169–79. doi: 10.1515/nanoph-2024-0331 (PMC11501062; doi:10.1515/nanoph-2024-0331)
Supplement: Supplementary file 1 — Supplementary Material Details [file j_nanoph-2024-0331_suppl_001.pdf]

Supporting Information

Multichannel full-space coding metasurface with linearly-circularly-polarized wavefront manipulation

Huiling Luo, Huanhuan Gao, Yanzhao Wang, Chaohui Wang, Fan Zhang, Yanzhang Shao, Tong Liu, Zhengjie Wang, and He-Xiu Xu*

Air and Missile Defense College, Air Force Engineering University, Xi'an 710051, China

*Corresponding Author

E-mail: hxxuellen@gmail.com (He-Xiu Xu)

KEYWORDS: Multichannel coding metasurface, full space, wavefront control, shared aperture, linearly-circularly-polarized

1. Comparison for coupling level of the meta-atom with and without metallic rings in the top and bottom layers

Internal crosstalk in a multichannel metasurface interferes with the performance of both reflected and transmitted waves, even reducing efficiency. Therefore, a low crosstalk meta-atom is essential to achieve independent EM modulation in completely separate channels. We provide comparison for coupling level of the meta-atom with and without metallic rings in two cases (β_1 and β_2 are fixed as 25° and 160°, respectively; β_1 and β_2 are fixed as 160° and 160°, respectively) to further understand the role of the metallic ring in the meta-atom, see **Figure S1**. Figure S1a-d show reflection amplitude and phase response for backward and forward LCP wave excitation when top and bottom metallic rings are present in the meta-atom. When l_x and l_y changes, the co-polarization transmissive amplitude of the coding status is higher than -1.5 dB, and all phase response remains unchanged at working frequency f_{c1}=17.1 GHz and f_{c2}=16.9 GHz. Meanwhile, **Figure S1e-h** below plot calculation results

without metallic rings for backward and forward LCP wave excitation at working frequency f_{w1}=16.9 GHz and f_{w2}=16.7 GHz, respectively. In sharp contrast, higher reflection amplitude and more consistent phase response of composite meta-atom with metallic rings are shown under forward and backward CP waves. Specifically, the phase shifts of the designed structure with metallic rings are extremely weak at operating frequencies, indicating that they are barely responding. In a word, all calculation results demonstrate that the designed full-space meta-atom with metallic ring exhibits excellent isolation characteristics, which provides a solid foundation for independent manipulation of EM waves in different channels.

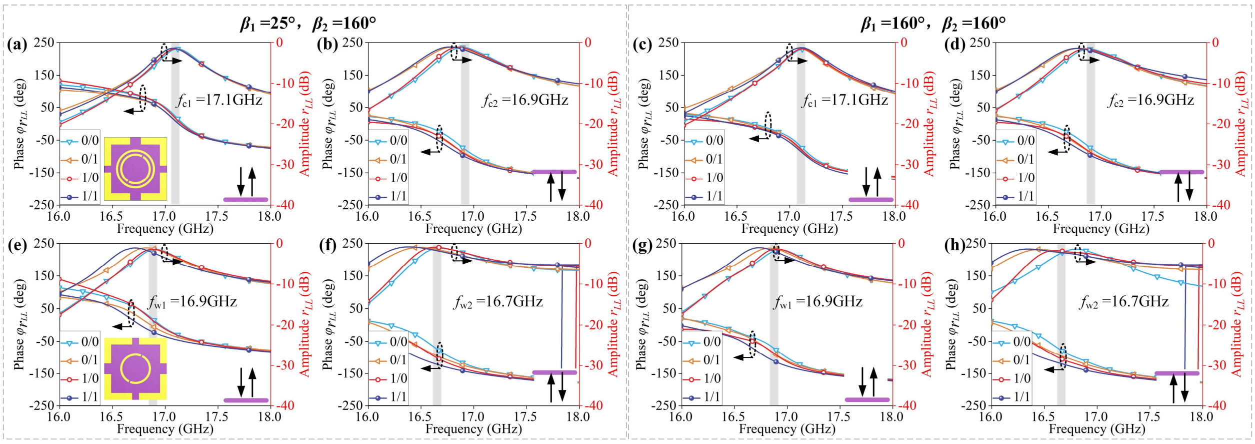

Figure S1 Coding states crosstalk. 2-bit coding sub-meta-atoms with reflection amplitude and phase response in the meta-atom with metallic rings, (a, b) when β_1 and β_2 are fixed as 25° and 160°, or (c, d) when β_1 and β_2 are fixed as 160° and 160°, respectively. 2-bit coding sub-meta-atoms with reflection amplitude and phase response in the meta-atom without metallic rings, (e, f) when β_1 and β_2 are fixed as 25° and 160°, or (g, h) when β_1 and β_2 are fixed as 160° and 160°, respectively.

2. The design process of coding pattern for dual-vortex beam

The discrete phase distribution of vortex beam is mathematically written as $\varphi_1 = l \bullet \arctan(y/x)$, where l is a topological charge of arbitrary integer, and denotes a range of unlimited orthogonal eigenstates, see **Figure S2a**. To devise dual-vortex beams (F_1) of 2-bit coding modes, the convolution operation of coding pattern carrying OAM mode l = 1 and a periodic coding pattern "00100010···" along the x direction is designed, and the design process of coding pattern is shown in **Figure S2**.

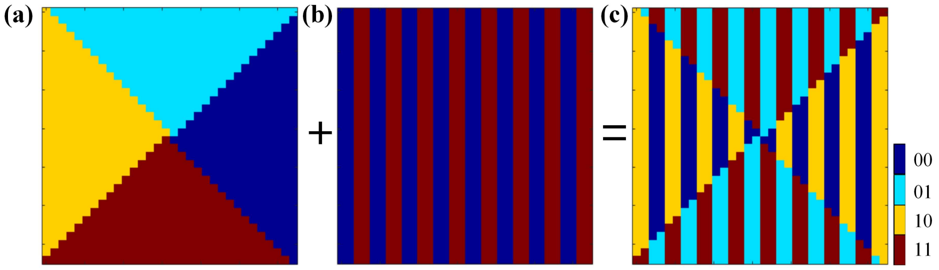

Figure S2 Coding pattern of dual-vortex beam. (a) 2-bit coding pattern carrying OAM mode l = 1. (b) A periodic coding pattern "00100010···" along the x direction. (c) The convolution operation result of coding patterns shown in (a) and (b).

3. Transmission phase calculation and coding pattern of polarization dependent beam splitting

To achieve a high-gain directional beam with low side-lobes, we analyze a schematic diagram of the beam deflection manipulation in **Figure S3**, where the plane wave is replaced by a feeding horn to excite the designed metasurface. According to the array theory, supposing that a beam is generated in a specified direction by the designed metasurface, then the local transmission phase should satisfy equation:

$$\phi_{n,m}(\theta,\varphi)=\phi_0+k_0S_{n,m}-k_0(x_{n,m}\sin\theta\cos\varphi+y_{n,m}\sin\theta\sin\varphi) \tag{S1}$$

where ϕ_0 is an arbitrary initial phase, k_0 is the wave number in the free space at target frequency f_0, $S_{n,m}$ is the distance transmission from the (n, m)th meta-atom to the feed source, $\phi_{n,m}(\theta, \varphi)$ is the calculated transmission phase of the (n, m)th meta-atom, θ and φ are the elevation angle and the azimuth angle of the designed beam, respectively, $x_{n,m}$ and $y_{n.m}$ are the coordinates of the (n, m)th meta-atom.

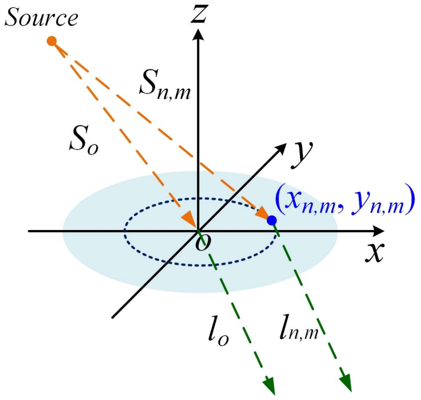

Figure S3 Schematic diagram of the proposed transmissive metasurface.

In our design, ϕ_0 is set to 0°, and the position of feed source is fixed above midpoint of the metasurface with a distance of F = 150 mm, which is beneficial for suppressing level of side-

lobes and achieving a high-gain beam. Assuming that the deflection angle is θ_x (θ_y) in the x (y) direction, i.e. φ = 0°, the phase distribution is described as follows:

$$\phi_x(\theta_x)=k_0 S_{n,m} - k_0 \cdot x_{n,m} \sin\theta_x \tag{S2}$$

$$\phi_y(\theta_y)=k_0 S_{n,m} - k_0 \cdot y_{n,m} \sin\theta_y \tag{S3}$$

where $S_{n,m}=\sqrt{x_{n,m}^2 + y_{n,m}^2 + F^2}$. Since the designed meta-atom of the transmissive metasurface has only two states, the continuous phases $\phi_{n,m}(\theta, \varphi)$, $\phi_x(\theta_x)$ and $\phi_y(\theta_y)$ have to be discretized. To achieve polarization beam splitting, **Figure S4** shows the process of digital code. To distinguish from dynamic phases, we deliberately apply different phase gradients under normal illumination of x- and y- LP waves. Particularly, we encode the coding sequence of deflection angle with θ_y = -30° along y-direction for x-LP wave, while deflection angle with θ_x = -45° along x-direction for y-LP wave, as illustrated in **Figure S4a,b**, respectively. Ultimately, **Figure S4c** shows coding pattern of designed polarization beam splitter by convolution operation.

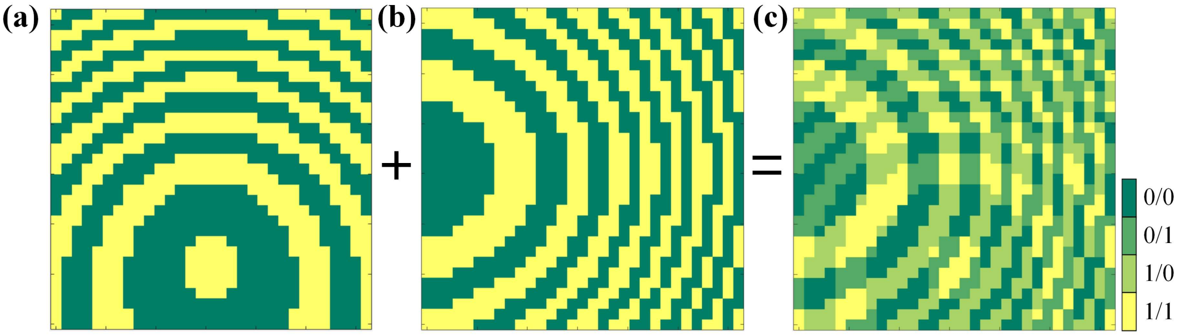

Figure S4 Designed process of polarization dependent beam splitting. (a, b) Coding pattern of deflection angle with θ_y = -30° and deflection angle with θ_x = -45°, respectively. (c) coding pattern of polarization beam splitting.

4. FDTD calculated field intensity of Bessel beam at *x-z* planes

To further evaluate the performance of Bessel beam, **Figure S5** presents FDTD calculated field intensity on x-z planes of z = 200, 350, and 500 mm away from the coding metasurface, when the meta-structure is illuminated by a normally incident plane wave under CP mode along +z axis at 16.9 GHz.

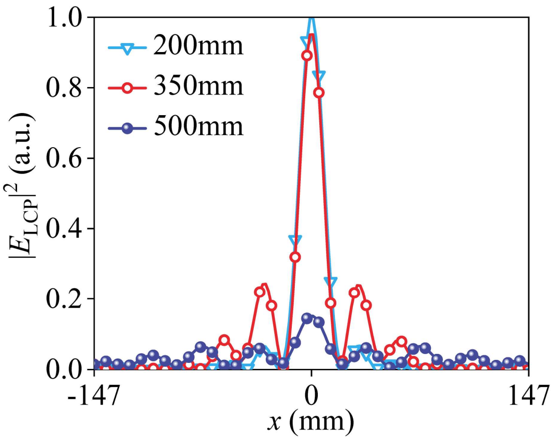

Figure S5 FDTD-calculated field intensity on x-z planes of z = 200, 350 and 500 mm away from the coding metasurface.

5. Photograph of fabricated sample

To verify the feasibility of the proposed strategy, a prototype composed of 32 × 32 meta-atoms is fabricated, as shown in **Figure S6**. The sample size is 288 × 288 mm^2. From top and bottom views of the structure, it can be observed that four "L" type patches are presented as diagonal crossbar patches in the metasurface.

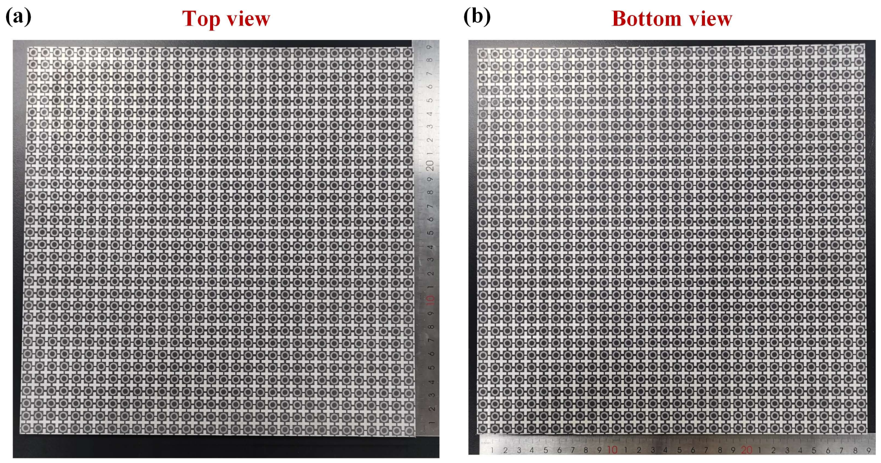

Figure S6 Photograph of the fabricated sample of the proposed multichannel full-space coding metadevice with the top and bottom panels as its two faces.

6. Transmission radiation patterns under illumination of *x*- and *y*-LP waves at 11.2 GHz

Except for 45°-LP wave case presented in **Figure 6c,d**, two cases are considered under the normal incidence of x- and y- LP waves for a systemic investigation, see Figure S7. As shown in **Figure S7a**, it can be observed that the transmission beam is deflected to the y-direction with θ_1 = -30° when the incident E-field is along x-direction at 11.2 GHz, showing an excellent

agreement with the theoretical prediction according to (S3). **Figure S7b** illustrates the radiation pattern when the metasurface is excited by the *y*-LP wave. The deflected beam is observed clearly observed with θ_2 = -45°, which is consistent with the theoretical prediction for a second time. The efficiency is simulated as 90.3 % and 87.9 % under illumination of *x*- LP wave, and measured as 88.1 % and 85.3 % under illumination of *y*- LP wave, respectively.

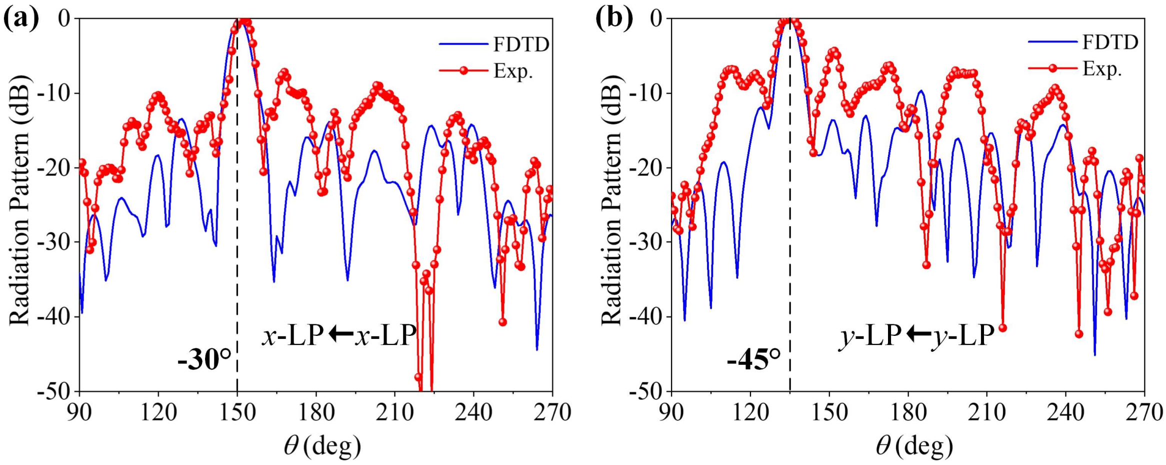

Figure S7 Far-field experimental demonstration of full-space coding metadevice. FDTD simulated and measured transmission radiation patterns under illumination of (a) *x*- LP and (b) *y*- LP waves at 11.2 GHz.
